# RT-DETR-SoilCuc: detection method for cucumber germinationinsoil based environment

**DOI:** 10.3389/fpls.2024.1425103

**Published:** 2024-08-22

**Authors:** Zhengjun Li, Yijie Wu, Haoyu Jiang, Deyi Lei, Feng Pan, Jinxin Qiao, Xiuqing Fu, Biao Guo

**Affiliations:** ^1^ College of Artificial Intelligence, Nanjing Agricultural University, Nanjing, China; ^2^ College of Engineering, Nanjing Agricultural University, Nanjing, China; ^3^ Institute of Mechanical Equipment, Xinjiang Academy of Agricultural and Reclamation Science, Shihezi, China; ^4^ Cotton Research Institute, Xinjiang Academy of Agricultural and Reclamation Sciences, Shihezi, China

**Keywords:** RT-DETR, soil-based environment, cucumber germination, germination rate, salt tolerance

## Abstract

Existing seed germination detection technologies based on deep learning are typically optimized for hydroponic breeding environments, leading to a decrease in recognition accuracy in complex soil cultivation environments. On the other hand, traditional manual germination detection methods are associated with high labor costs, long processing times, and high error rates, with these issues becoming more pronounced in complex soil–based environments. To address these issues in the germination process of new cucumber varieties, this paper utilized a Seed Germination Phenotyping System to construct a cucumber germination soil–based experimental environment that is more closely aligned with actual production. This system captures images of cucumber germination under salt stress in a soil-based environment, constructs a cucumber germination dataset, and designs a lightweight real-time cucumber germination detection model based on Real-Time DEtection TRansformer (RT-DETR). By introducing online image enhancement, incorporating the Adown downsampling operator, replacing the backbone convolutional block with Generalized Efficient Lightweight Network, introducing the Online Convolutional Re-parameterization mechanism, and adding the Normalized Gaussian Wasserstein Distance loss function, the training effectiveness of the model is enhanced. This enhances the model’s capability to capture profound semantic details, achieves significant lightweighting, and enhances the model’s capability to capture embryonic root targets, ultimately completing the construction of the RT-DETR-SoilCuc model. The results show that, compared to the RT-DETR-R18 model, the RT-DETR-SoilCuc model exhibits a 61.2% reduction in Params, 61% reduction in FLOP, and 56.5% reduction in weight size. Its mAP@0.5, precision, and recall rates are 98.2%, 97.4%, and 96.9%, respectively, demonstrating certain advantages over the You Only Look Once series models of similar size. Germination tests of cucumbers under different concentrations of salt stress in a soil-based environment were conducted, validating the high accuracy of the RT-DETR-SoilCuc model for embryonic root target detection in the presence of soil background interference. This research reduces the manual workload in the monitoring of cucumber germination and provides a method for the selection and breeding of new cucumber varieties.

## 1 Introduction

Cucumber (*Cucumis sativus* L.), widely loved for its fresh, crispy texture, rich nutritional value, and powerful health benefits, is one of the most widely consumed fruits in the world (Wang et al., 2017). Several bioactive compounds found in cucumbers, including cucurbitacins, are believed to have potential anti-diabetic, lipid-lowering, and antioxidant activities (Mukherjee et al., 2012). With the continuous development of the edible and medicinal value of cucumbers, rapid breeding research for cucumbers is also advancing (Hui et al., 2023). Germination rate is an important indicator for evaluating the quality of cucumber varieties. Evaluating the germination rate of cucumbers under salt stress in soil-based environments is more closely aligned with the actual growth conditions of the seeds. This process can help identify cucumber varieties with stronger resistance to stress, aiding in the selection of more resilient cucumber cultivars. On one hand, traditional methods for assessing seed germination rely on manual judgment, which has the disadvantages of high cost, long time consumption, and high error rates (Jahnke et al., 2016). On the other hand, existing deep learning–based seed germination detection technologies are often optimized for hydroponic cultivation environments and may face challenges in accurately identifying germination in soil images with stronger interference (Jiang et al., 2023; Fu et al., 2022). Lastly, due to the limited performance of agricultural production equipment, practical germination detection technologies need to consider the lightweight nature of germination detection models. To address the limitations of current germination detection methods and take into account the deployment requirements in low-computing environments, this paper proposes a lightweight, cost-effective, automated, and high-throughput method for detecting cucumber seed germination in soil-based environments.

In the field of machine learning, classical machine learning methods have been widely used for automated analysis of seeds (de Medeiros et al., 2020a; Fu et al., 2021). For example, Zhao et al. (2022) and Khatri et al. (2022) successfully achieved the measurement of key dynamic traits of wheat (such as root length) using supervised machine learning algorithms such as Support Vector Machine and K-Nearest Neighbors. Shetty et al. (2011) employed a supervised classification method to predict the vigor of carrot or radish seeds using near-infrared spectroscopy. de Medeiros et al. (2020b) combined X-ray analysis with an linear discriminant analysis (LDA) machine learning model to achieve an average germination detection accuracy of 94.36% for leprosy tree seeds. Joosen et al. (2010) proposed a software package for assessing Arabidopsis seed germination. Yuan et al. (2016) and Dang et al. (2020) used red, green and blue (RGB)-transformed images for seedling recognition, but the recognition rate was only 50%, making it difficult for practical production applications. In summary, early machine learning–based crop phenotyping analysis methods often require the development of algorithms tailored to specific environments, with narrow applicability and low robustness. The algorithm design process is complex, and these methods typically operate only in specialized environments to identify specific crop features, lacking the flexibility for deployment and adaptability to environments with strong interference. Furthermore, their accuracy is relatively low, making it challenging to meet practical production demands. To overcome these limitations, convolutional neural networks have been more widely applied in crop phenotype analysis, as they can extract target features through multi-layer convolution and pooling operations and possess high robustness and real-time performance in deep learning models [e.g., Real-Time DEtection TRansformer (RT-DETR) and You Only Look Once (YOLO)].

In the field of deep learning, the YOLO algorithm has become the most widely used algorithm in the intelligent breeding field of agriculture due to its combination of fast detection speed, detection accuracy, and ease of use and improvement (Yanhua et al., 2022; Redmon et al., 2016). Many scholars have developed more accurate algorithms based on YOLO for specific environments and varieties to address different application scenarios (Li et al., 2024). For example, Yao et al. (2024) and Kundu et al. (2021) utilized the improved YOLOv7 algorithm and YOLOv5 algorithm, respectively, for wild rice germination detection and maize seed classification. The abovementioned germination detection algorithms based on YOLO typically operate in hydroponic environments where the images are captured against a solid-colored non-woven fabric background. The solid-colored background in hydroponic environments minimizes background interference, and the special optimizations implemented in these algorithms are often geared towards accurately detecting small embryo targets rather than combating background interference. While these algorithms perform well in hydroponic environments, the complex background interference in soil-based environments, which is closer to actual production settings, can significantly impact their recognition performance.

Although the YOLO series algorithms have been widely used in agricultural production experiments, they still have significant drawbacks. YOLO tends to generate numerous redundant bounding boxes during runtime, which need to be filtered out using non-maximum suppression (NMS) in post-processing. The hyperparameters of NMS can significantly impact the accuracy and speed of YOLO (Lv et al., 2023). This can lead to poor detection of small objects (Jiang et al., 2023) and a large number of parameters. Dai et al. (2021) introduced a dynamic encoder in the DETR model to approximate the attention mechanism of the Transformer encoder, improving the DETR model’s ability to recognize features of small objects. Dai et al. (2021) proposed the unsupervised pre-training (UP)-DETR model, which exhibits faster convergence in object detection, to some extent addressing the issue of lengthy training time in DETR. Unlike the common hydroponic environment in laboratories, although soil-based cultivation is closer to actual production, irregular small soil particles scattered around seeds or adhering to tiny newly embryonic roots pose a significant challenge for the YOLO model, which is not adept at recognizing small objects. To achieve high accuracy and recall in soil-based cultivation while considering the cost and training time, it is advisable to abandon the use of YOLO and instead opt for lightweight models in the DETR series, such as the real-time end-to-end object detection model, RT-DETR, as the baseline model for improvement (Lv et al., 2023). Compared to YOLO, it has a mixed encoder that can effectively handle multi-scale features and Intersection over Union (IoU)-aware query selection, enabling RT-DETR to achieve faster speed than YOLO at the same level of accuracy, thus outperforming YOLO in the field of small object detection.

While RT-DETR has advantages in recognition accuracy compared to YOLO of similar scale, there are still limitations in cucumber seed germination detection in soil-based environments. Firstly, unlike hydroponic environments, in soil-based, cucumber seeds tend to burrow into the soil during germination, leading to the seeds themselves or the radicles being covered by soil. Radicles emerging from another position after burrowing into the soil also easily result in repeated identification of the same target. To address these complex scenarios, more targeted image data need to be collected. The time span of cucumber germination is 3 to 4 days, and manually annotating a large amount of image data generated during this process is extremely time-consuming. Therefore, to achieve better training results with a limited annotated training set, the dataset needs to be augmented online to increase the training data volume. On the other hand, cucumber seeds are small in size, and the radicles that emerge during germination are also fine. In soil-based environments, compared to hydroponic environments, the background of cucumber seed images may contain more interfering objects, including but not limited to stones with colors similar to cucumber seeds and wooden fibers similar in length to radicles. Despite RT-DETR having superior small target resolution capabilities compared to YOLO, distinguishing fine radicles from small interfering objects in the soil remains challenging, necessitating enhancement of the recognition capabilities of the RT-DETR model for soil-based environments. Additionally, the parameter size of the RT-DETR model is large. To reduce deployment complexity and improve inference speed, efforts are made to reduce its parameter size and computational load through additional lightweight design. This paper proposes a RT-DETR-SoilCuc model specifically designed for cucumber seed germination detection in soil-based, using the RT-DETR-R18 model with a smaller parameter size in the RT-DETR series as the baseline model (Lv et al., 2023).

To address the abovementioned issues, the main contributions are as follows:

1. To ensure the model’s recognition accuracy in the face of complex soil interference, we introduce online image augmentation techniques based on the Albumentations library. This approach not only reduces the cost of manual annotation but also enhances the efficiency of model training in limited datasets and improves the model’s adaptability to complex environments.
2. By incorporating the Adown downsampling operator and the Generalized Efficient Lightweight Network (GELAN) module derived from YOLOv9 into the backbone of the model, we have successfully enhanced the model’s recognition accuracy while reducing its computational complexity. This ensures that the model can be deployed in scenarios with limited hardware resources and achieves seamless upgrades without compromising performance.
3. Introducing the Online Convolutional Re-parameterization (OREPA) mechanism into the model’s convolutional computation allows flexible weight transformation during model training and enhances the model’s ability to identify small targets with uncertain embryonic root positions.
4. The introduction of the Normalized Gaussian Wasserstein Distance (NWD) loss function specifically designed to enhance the recognition of small targets, combined with the previous improvements, strengthens the model’s ability to identify embryo features in the presence of soil background interference.
5. Validating the practical use of the model through a salt-alkali tolerance experiment on cucumber seeds: The model is used to automatically analyze seed germination, and in combination with germination rate and germination index, successfully evaluates the salt-alkali tolerance of the cucumber variety, thereby verifying the practical value of the model.

The remaining sections of this paper are organized as follows. The second section discusses the data collection equipment and methods, the process of image data collection, data augmentation, the structure of RT-DETR-SoilCuc and its improvement modules, model evaluation metrics, and seed germination vigor evaluation metrics. The third section explains the results and discussions, including training environment and parameter settings, results and discussions of model ablation experiments and comparative experiments, and experimental verification of the model’s effectiveness in detecting salt and alkali tolerance of cucumber seeds in soil. The fourth section provides a summary, limitations, and future work.

## 2 Materials and methods

### 2.1 Data collection equipment

**Figure 1:**
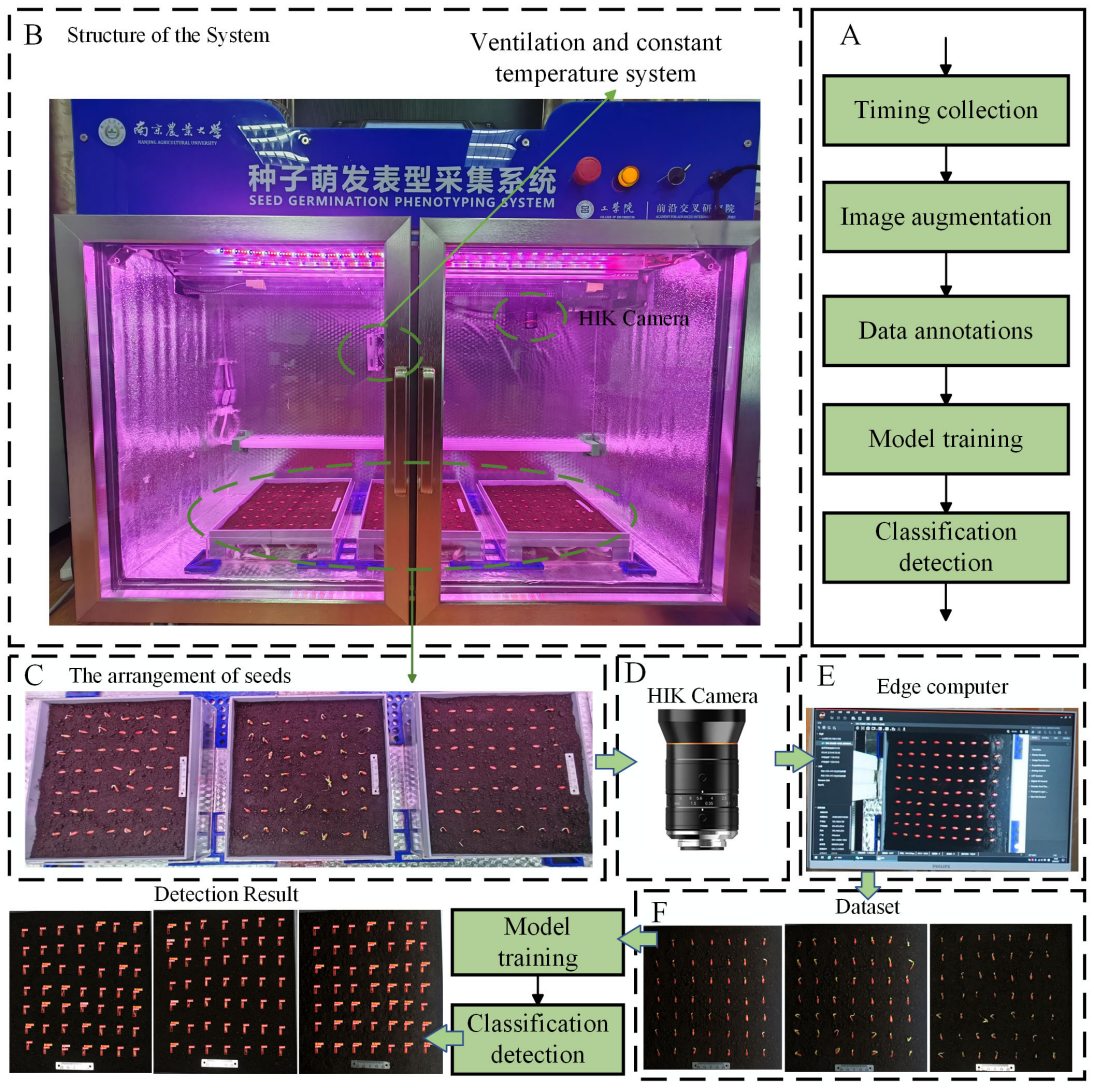
Seed Germination Phenotyping System. **(A)** Schematic diagram of model training process. **(B)** Structure of the system. **(C)** Schematic diagram of seed placement. **(D)** Data acquisition camera. **(E)** Edge computer. **(F)** Schematic of the acquired images.

We conducted cucumber seed germination experiments using a Seed Germination Phenotyping System. The system comprises a seed germination incubator and an image acquisition device, as shown in **Figure 1B**. The dimensions of the incubator are 1,120 mm in length, 380 mm in width, and 860 mm in height, with internal dimensions of 1,060 mm in length, 320 mm in width, and 800 mm in height (manufacturer: Henan Greentech Electric Technology Co., Ltd.). It is equipped with a hot air circulation system that relies on two sets of Tp-100 thermocouples on the inner side of the incubator to monitor the temperature. When the temperature falls below the preset level, the hot air circulation system operates to raise the temperature. If the temperature exceeds the upper limit, then the system stops to maintain a constant temperature environment. The temperature range can be adjusted between 5°C and 50°C to meet the germination temperature requirements of different seeds. Additionally, the box has an LED lighting system and can accommodate three 25 cm × 25 cm culture dishes, each of which can cultivate 49 seeds simultaneously, as shown in **Figure 1C**. To avoid reflections from the acrylic material affecting image acquisition, the culture dishes are customized using Polyethylene terephthalate-I (PETG) material through 3D printing technology. A horizontal guide rail is installed at the top of the incubator, with a stepper motor and an HIK Vision industrial camera mounted on the rail. The camera model is MV-CS060-10GC, equipped with a 12-mm focal length lens (model MVL-HF1228M-6MPE, supplier: Suzhou Youxin Zeda Co.), positioned 40 cm away from the culture dish. The stepper motor provides power to move the camera along the guide rail inside the incubator at a speed of 0 mm to 50 mm per second, covering a range of 860 mm, to capture high-resolution images of specific culture dishes. The camera communicates with the host through a GigE gigabit interface, and the image resolution is 2,592 × 2,048 pixels. Users have the option to adjust the camera’s focal length, shooting interval, and apply pre-cropping of images through the software interface on the host, as shown in **Figure 1E**. The final images can be viewed in the “data” folder on the host for the next step of dataset annotation. The annotated dataset, as shown in **Figure 1F**, is used for model training. The model training process is illustrated in **Figure 1A**, and the final model recognition results are shown in **Figure 1**.

### 2.2 Data collection and preprocessing

#### 2.2.1 Germination protocol and data collection

**Figure 2:**
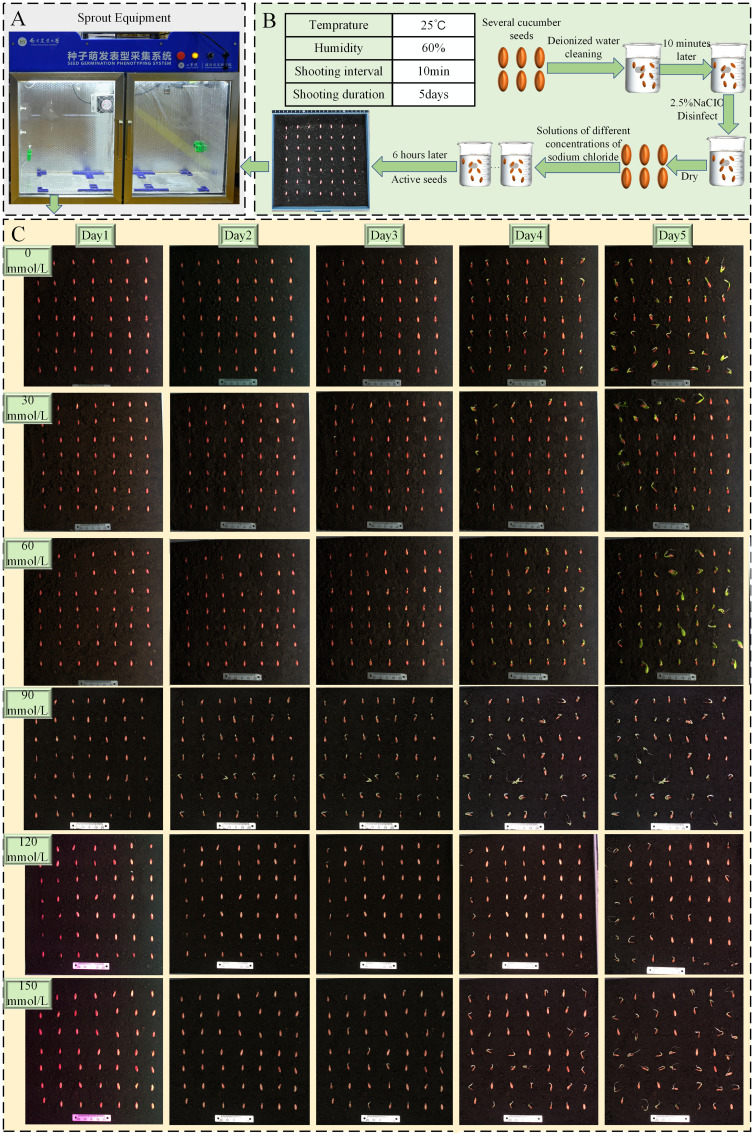
**(A)** Sprout equipment. **(B)** Experimental flowchart. **(C)** Schematic of cucumber seed growth process.

We conducted experiments using the Zhi Lv 0116 (supplier: Nanjing Lvling Seed Industry Co., Ltd.) variety combined with pure substrate soil. The pure substrate soil that has been sterilized at high temperatures does not contain harmful pathogens that affect cucumber germination. It has a darker color and fewer impurities, making it easier to distinguish seeds from the soil background. By utilizing the Seed Germination Phenotyping System (as shown in **Figure 2A**), we maintained the experimental temperature at 25°C, ensuring full-day sunlight and suitable humidity (Guadalupe et al., 2013). A total of 500 undamaged and plump cucumber seeds were selected for the experiment, as shown in **Figure 2B**. The experiment was divided into two parts: a non-stress control experiment and a salt stress experiment. The salt stress experiment involved salt concentrations ranging from 30 mmol/L to 150mmol/L, with experiments conducted at intervals of 30 mmol/L, totaling five sets of experiments (Deinlein et al., 2014). The non-stress control experiment was conducted six times, spanning 5 days, resulting in a collection of 2,102 images. The salt stress experiment was also repeated six times for each group, lasting 5 days, and yielding a total of 3,991 images, with images at key time points shown as an example in **Figure 2C**. All images were saved in.jpg format with a resolution of 1,840 × 1,800 pixels after pre-cropping on the host.

#### 2.2.2 Data preprocessing

**Figure 3:**
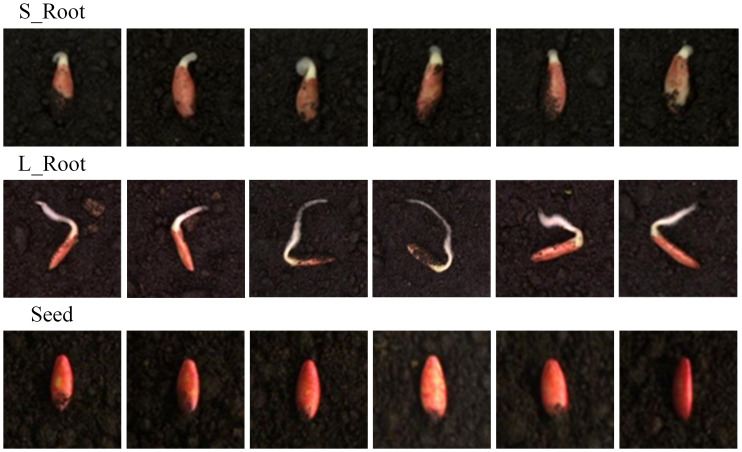
Schematic of short root and long root cucumber seeds.

To ensure the efficiency of later training, we manually selected early germination images from a total of 6,093 pictures and removed blurry images caused by exposure failures. This resulted in the extraction of 1,000 high-quality early germination seed images. We used LabelImg to manually annotate these 1,000 images, thereby establishing a dataset. To enrich the variety of data and improve training accuracy, roots shorter than the length of the seed itself were labeled as “SROOT,” whereas roots longer than the seed itself were labeled as “LROOT.” Seed vitality was determined on the basis of whether the seed could germinate and the length of the root. The annotation criteria are shown in **Figure 3**.

**Figure 4:**
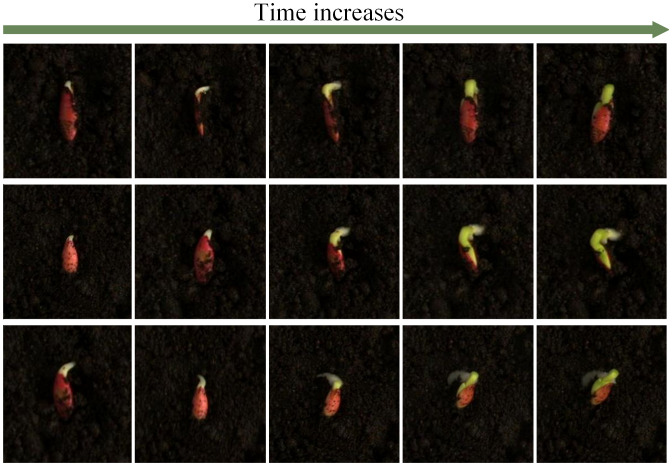
Root schematic diagram for drilling into soil.

The dataset will be divided into training, testing, and validation sets in a 7:2:1 ratio. Considering the traditional offline image augmentation methods, expanding images from the original dataset may appear in both the testing and training sets, potentially leading to artificially inflated model accuracy. Additionally, in soil-based seed cultivation environments, the radicles of seeds may penetrate the soil or become covered in soil, as shown in **Figure 4**. Small target radicles covered by soil in high-intensity data augmentation, such as pixelation or adding mosaics, may result in the loss of some information, further negatively impacting the model training effectiveness. To address this issue, this study will employ online data augmentation based on the Albumentations library (Buslaev et al., 2020). This form of data augmentation will apply random augmentation within preset parameter ranges during the model training process, ensuring that each batch of trained images undergoes different data augmentation. Compared to traditional offline data augmentation methods, which can only use a fixed method to expand the dataset, the online data augmentation approach generates almost limitless samples.

**Figure 5:**
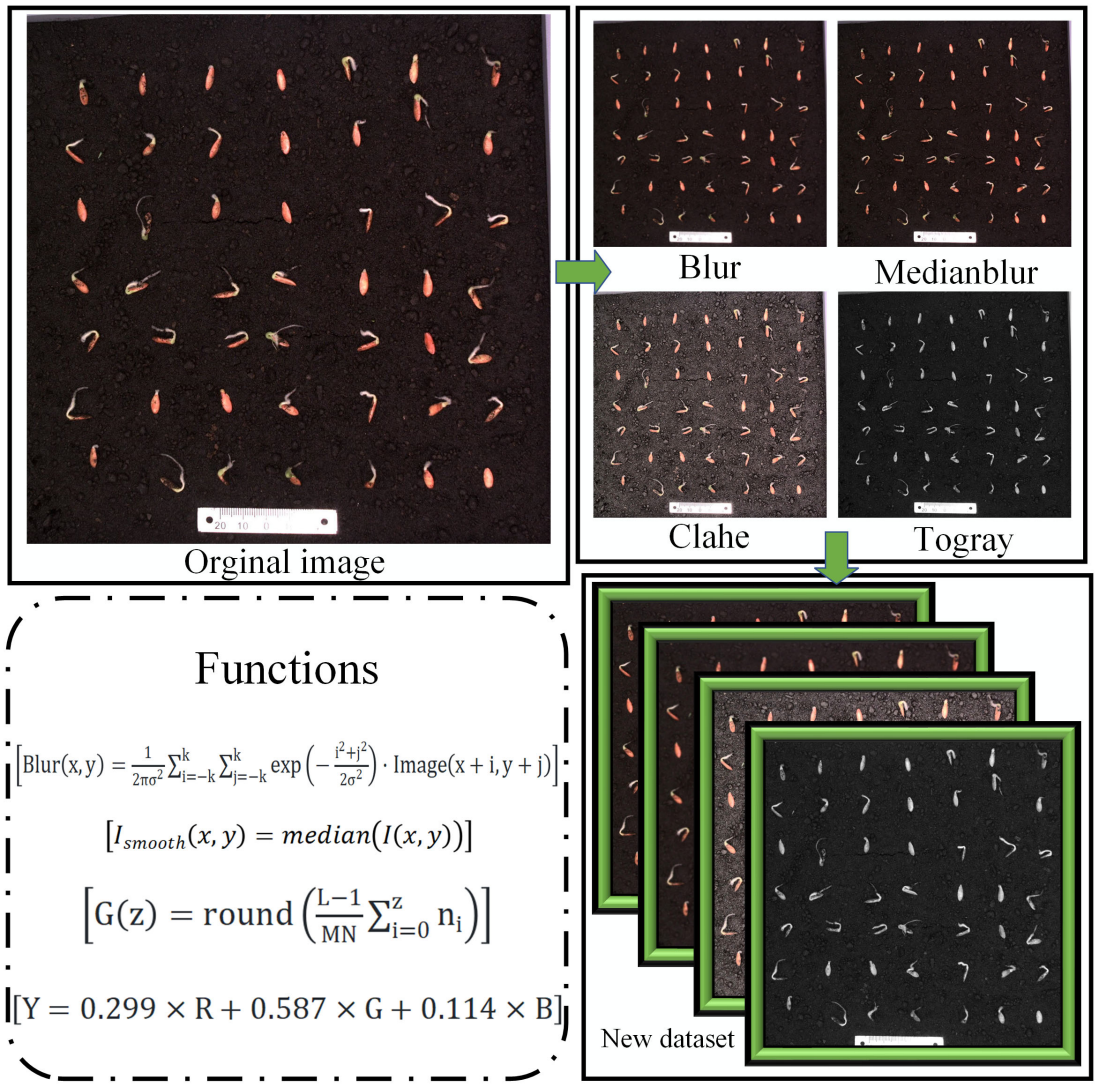
Schematic diagram of online image augmentation process.

Considering that online data augmentation may increase model training time, this paper adopts four relatively mild and simple image enhancement functions for data augmentation (1) Blur: applies random blurring to the entire image with a blurring probability set at 1%, simulating image blurring caused by camera shake. (2) Medianblur: uses median filtering to take the average of surrounding pixels, reducing image noise and enhancing training effectiveness. (3) Clahe (Contrast Limited Adaptive Histogram Equalization): randomly adjusts the contrast of the image to enhance the model’s recognition capability under varying lighting conditions. (4) Togray: converts the image to a grayscale image to reduce GPU resource usage. The results of the image processing are shown in **Figure 5**.

### 2.3 Design for RT-DETR-SoilCuc

The RT-DETR-SoilCuc model proposed in this paper is an improvement based on the lightweight RT-DETR-R18 model (Lv et al., 2023). The baseline model has been further lightweighted, and, for the purpose of facilitating model deployment, it has been modified from using the PaddlePaddle library to using the ultralytics library for deployment. This allows RT-DETR-SoilCuc to run in the YOLO environment, making it easier to use, deploy, and improve. Similar to traditional neural networks, RT-DETR-SoilCuc consists of three main parts: the backbone, the neck, and the decoder and head parts. The backbone first extracts shallow and effective information from the image through two consecutive convolution operations. In the P3 to P5 layers, the use of the downsampling operator ADown and the combination of two modules based on GELAN and OREPA, OREPANCSPELAN4, can feed the mid-training information back to the training loop, making the model more efficient while computing lightweight, and able to identify deep semantic features of small targets. In the decoder and head parts, RT-DETR-SoilCuc has a new group attention mechanism, which can distribute attention calculation to two attention heads to improve training efficiency.

**Figure 6:**
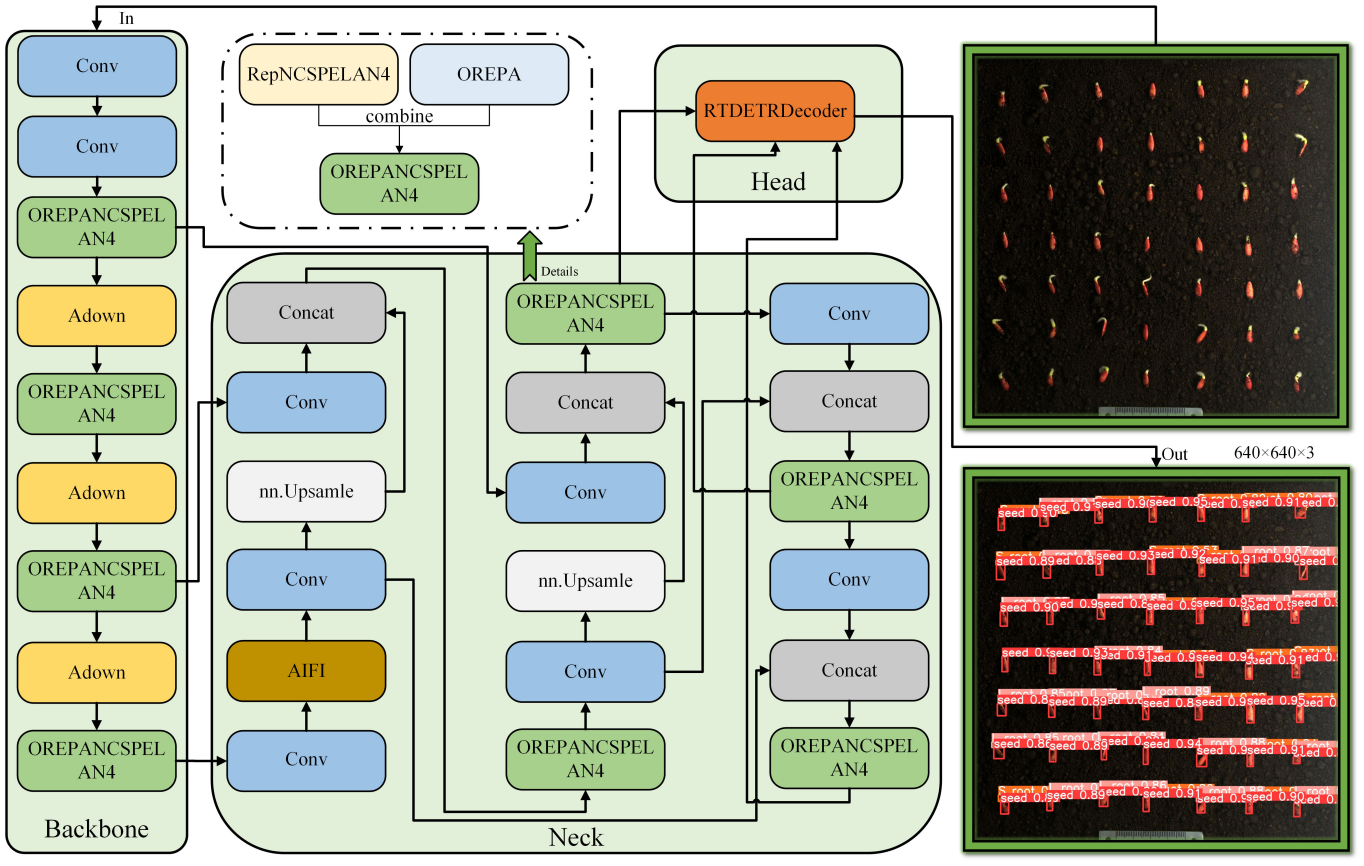
RT-DETR-SoilCuc detector structure scheme.

Compared to RT-DETR-R18, this paper makes the following improvements: (1) In the backbone part, we added the downsampling operator Adown from the latest YOLOv9, which uses average pooling to extract feature map sizes, reducing the number of model parameters and making the model more lightweight. At the same time, Adown can fuse its parameters into the convolutional layer during the inference stage, making the model more lightweight and efficient. (2) The RepNCSPELAN4 module from YOLOv9 replaces the Repc3 module in RT-DETR-R18. It is more lightweight and can provide a more versatile and efficient network to adapt to complex training tasks. (3) To avoid a decrease in accuracy after lightweighting the model, we incorporated OREPA into the RepNCSPELAN4 module to reduce training complexity and improve model accuracy. (4) We added the NWD loss function specifically designed to enhance the semantic extraction capability of small targets, aiming to improve the recognition of fine embryonic root features. The improved model structure is shown in **Figure 6**.

#### 2.3.1 Adown

**Figure 7:**
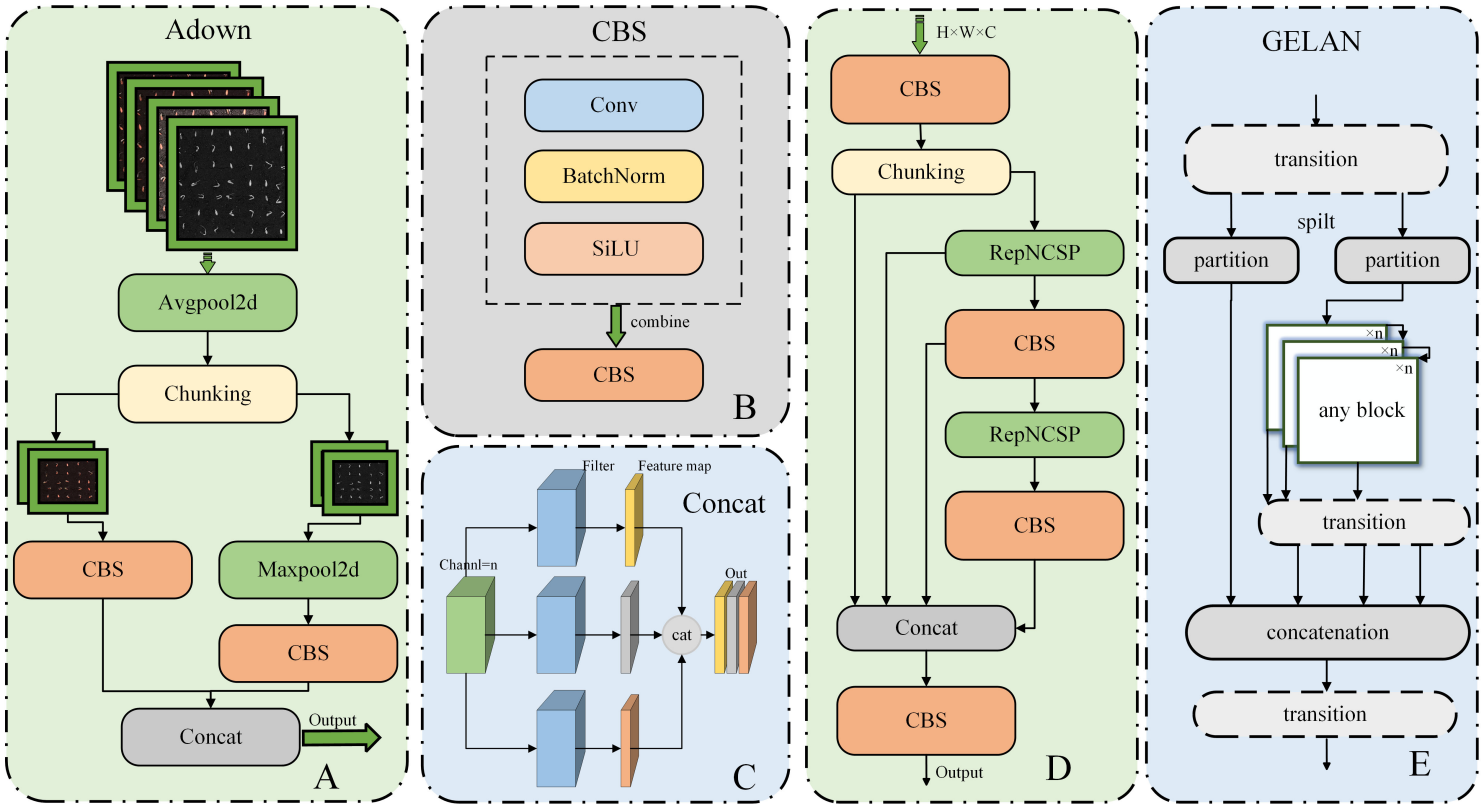
Structure diagram of Adown in RT-DETR-SoilCuc. **(A)** Adown module. **(B)** CBS module. **(C)** Concat module. **(D)** RepNCSPELAN4 module structure. **(E)** GELAN schematic.

The module is the downsampling module in YOLOv9, and we will insert this module between the convolution operations in the backbone of RT-DETR-R18. The structure of Adown and its sub-modules is shown in **Figure 7**, where chunking is the segmentation operation performed on the input dataset.

Different from the traditional downsampling module, which is typically a direct combination of max pooling and average pooling, Adown introduces two convolution operations (Wang et al., 2024). It first provides average pooling to average all the features in the image, helping to extract overall features. It then splits the channels into two parts. Each part undergoes a CBS operation and a max pooling operation. CBS is a combination of convolution, batch normalization, and activation function, which can extract the features within the image after average pooling, whereas max pooling retains the most significant features of each target. Finally, the two results are concatenated to combine feature maps of different scales in the channel dimension, expanding the tensor dimension to further extract deep semantic information. By adding this module, the model’s efficiency can be improved without significantly reducing accuracy.

#### 2.3.2 RepNCSPELAN4

RepNCSPELAN4 is derived from the new network architecture proposed in YOLOv9 called GELAN, as shown in **Figure 7**. It is a fusion of cross stage partial network (CSPNet) and efficient layer aggregation network (ELAN) designs (Wang et al., 2019). In the architecture of CSPNet, the input is divided into two parts through a transition layer and then processed separately through arbitrary computation blocks. These branches are then recombined (via concatenation) and passed through the transition layer again. On the other hand, ELAN adopts stacked convolutional layers, where the output of each layer is combined with the input of the next layer and then processed through convolution (Zhang et al., 2022).

The new neural network architecture, GELAN, combines the segmentation and recombination concepts of CSPNet and the hierarchical convolution processing approach of ELAN to improve the model’s performance and flexibility (Wang et al., 2024). It is designed considering lightweighting, inference speed, and accuracy to enhance overall performance. It incorporates the segmentation and recombination concepts of CSPNet to improve the model’s feature extraction and recombination capabilities. Additionally, it introduces the hierarchical convolution processing approach of ELAN in each part, further enhancing the model’s performance and adaptability.

Unlike previous architectures, GELAN not only uses convolutional layers but can also use any computation block, making the network more flexible and customizable according to different application requirements. This structure allows GELAN to support various types of computation blocks to better adapt to different computing requirements and hardware constraints. Furthermore, the optional modules and partitions of GELAN further increase the network’s adaptability and customizability. This design enables GELAN to better adapt to various application scenarios and hardware platforms, thus improving the model’s flexibility and performance. This also provides a breakthrough for further model improvement and will replace the ordinary convolution modules in RT-DETR as a computational module.

#### 2.3.3 OREPA

**Figure 8:**
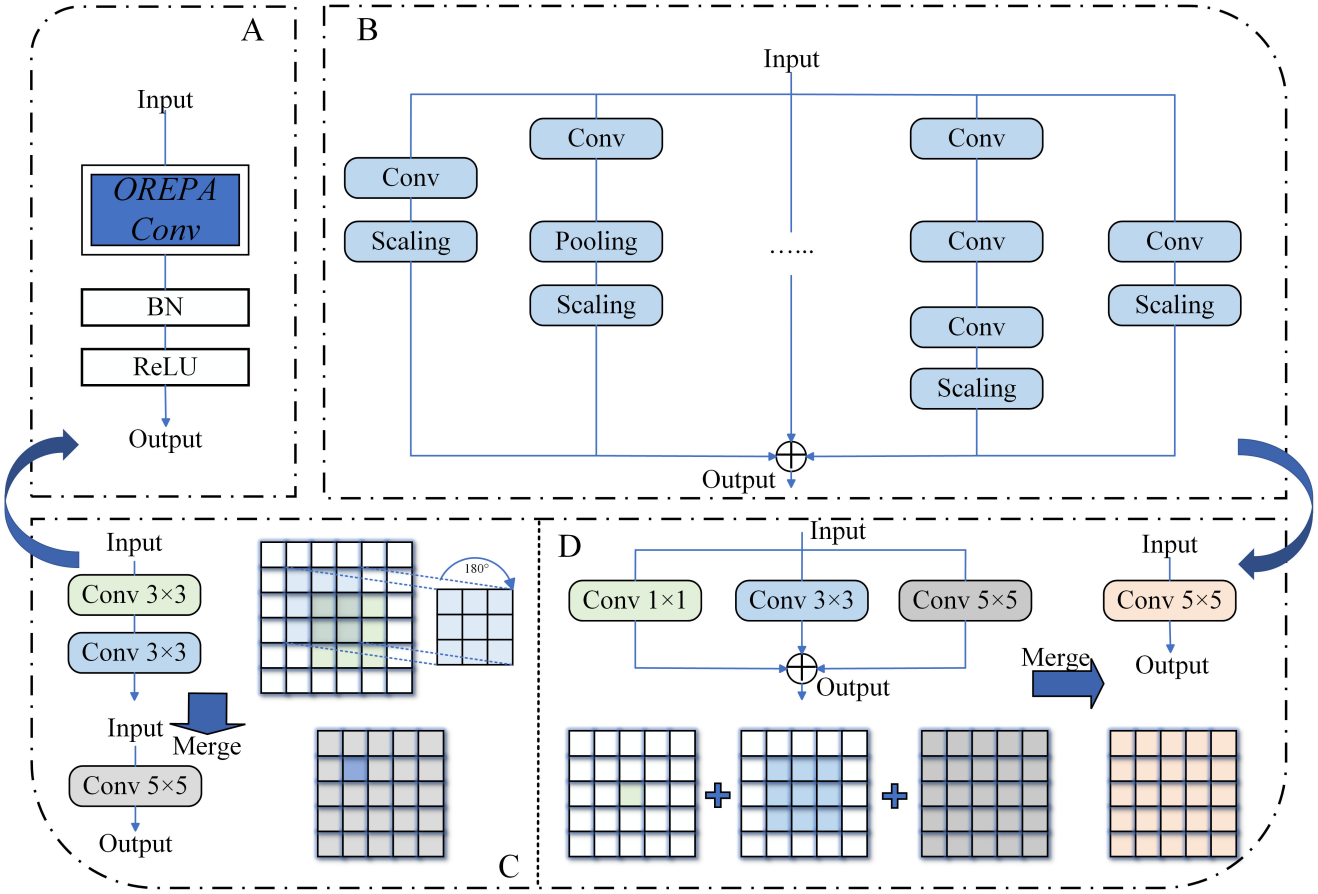
**(A)** OREPA module structure. **(B)** Block linearization schematic. **(C, D)** Block squeezing schematic, including sequential structure **(C)** and parallel structure **(D)**.

OREPA was proposed by Hu et al. at CVPR 2022 to overcome the limitations of traditional structural re-parameterization, which often trades training time for training accuracy (Hu et al., 2022). To achieve this goal, OREPA consists of two main components: a special linear scaling layer and module compression, as shown in **Figure 8**.

The linear scaling layer is used to optimize the online module and improve training efficiency. It allows the model to perform real-time refreshing of weights during training, enhancing the flexibility of the model while effectively extracting information. Additionally, stable weight flow further optimizes the model. The compression of the second-stage module refers to OREPA’s transformation of a large number of complex convolutional network blocks into a single convolutional layer through simple linear processing and structural simplification during training. This significantly reduces the parameter count and computation time during training, making the model easier to deploy. OREPA reduces the additional training overhead caused by a large number of intermediate modules and has minimal impact on model accuracy.

Leveraging the unique scalability of GELAN, we used class inheritance to integrate OREPA into RepNCSPELAN4, replacing two convolutional blocks. This approach reduces the computational load of the model backbone and filters out the interference of features related to the soil-based environment.

#### 2.3.4 NWD

A targeted loss function can improve model accuracy while reducing computational complexity. We will add the specialized loss function, NWD, in addition to retaining the original Giou loss function (Wang et al., 2021). The core idea of NWD lies in the design of multi-scale windows and the fusion of multi-size features. This allows NWD to use different-sized detection windows at different stages of training and dynamically adjust window sizes for different targets. NWD can also fuse feature maps at different levels, aiding in the extraction of more contextual information. However, NWD is not sensitive to targets of different sizes, making it more suitable for extracting similarities between small objects.


(1) $$NWD\left(N_{a},N_{b}\right)=exp\left(-\frac{\sqrt{W_{2}^{2}\left(N_{a},N_{b}\right)}}{C}\right)$$

Here, W_2_^2^ represents the second-order Wasserstein distance between N_a_ and N_b_, and C is a constant related to the characteristics of the dataset.

### 2.4 Evaluation metrics

#### 2.4.1 Model evaluation metrics

To evaluate the level of model lightweighting and its effectiveness in detecting cucumber seed germination in soil-based environments, we will use precision, recall, floating point operation (FLOP), and Params for model evaluation, where precision represents the accuracy of the model’s target prediction, mAP represents the average accuracy of all target detections, and recall represents the ratio of correctly predicted targets to the total targets. The closer these parameters are to 1, the better the model’s performance. It is worth noting that, for the detection performance of small embryonic roots, more attention should be paid to mAP@0.5-0.95 rather than mAP@0.5. The numbers following them represent the ratio of the detection box to the actual target. Clearly, for small targets, the requirement for the accuracy of the detection box selection is greater than that for larger targets. FLOP represents the computational complexity required by the model, and Params represents the level of model lightweighting. The lower these values, the lower the model’s training cost and the higher the level of lightweighting. An excellent and effective soil-based cucumber germination detection model should achieve high accuracy and high recall while ensuring a lightweight level. True positive (TP) refers to the count of cucumbers correctly identified by the model as germinated, whereas false positive (FP) and false negative (FN) indicate the number of cucumber seeds that are erroneously identified or missed by the model, respectively. The formulas for calculating each evaluation parameter are as follows:


(2) $$Precision=\frac{TP}{TP+FP}$$

(3) $$Recall=\frac{TP}{TP+FN}$$

(4) $$AP=\int_{0}^{1}P(R)dR$$

(5) $$mAP=\frac{\int_{q=1}^{Q}AP(q)}{n}$$

(6) $$FLOPS=2\times H\times W\left(C_{in}K^{2}+1\right)C_{\text{out }}$$

(7) $$Params=C_{\text{in }}\times K^{2}\times C_{\text{out}}$$

#### 2.4.2 Evaluation metrics of seed germination vigor

This paper primarily employs two metrics to assess the viability of cucumber seeds: germination rate and germination index. The germination rate is defined as the proportion of germinated seeds to the total number of seeds at a specific time, which is an intuitive reflection of seed vitality and is widely used in agricultural production to evaluate the germination potential of seeds. The germination index refers to the ratio of the total number of seeds germinated by a certain time to the total number of days for seed germination. This index can effectively assess the future vitality and growth potential of the seeds. By calculating these two indicators, the vitality of cucumber seeds in a soil-based environment can be accurately reflected, providing evaluation criteria for selecting superior genotypes of cucumber varieties. The specific formulas are shown in the following:


(8) $$Germination\;rate=\frac{N_{t}}{N}\times 100\%$$

(9) $$Germination\;index=\sum \left(\frac{G_{t}}{D_{t}}\right)$$

## 3 Results and discussion

### 3.1 Training environment and hyperparameter settings

**Figure 9:**
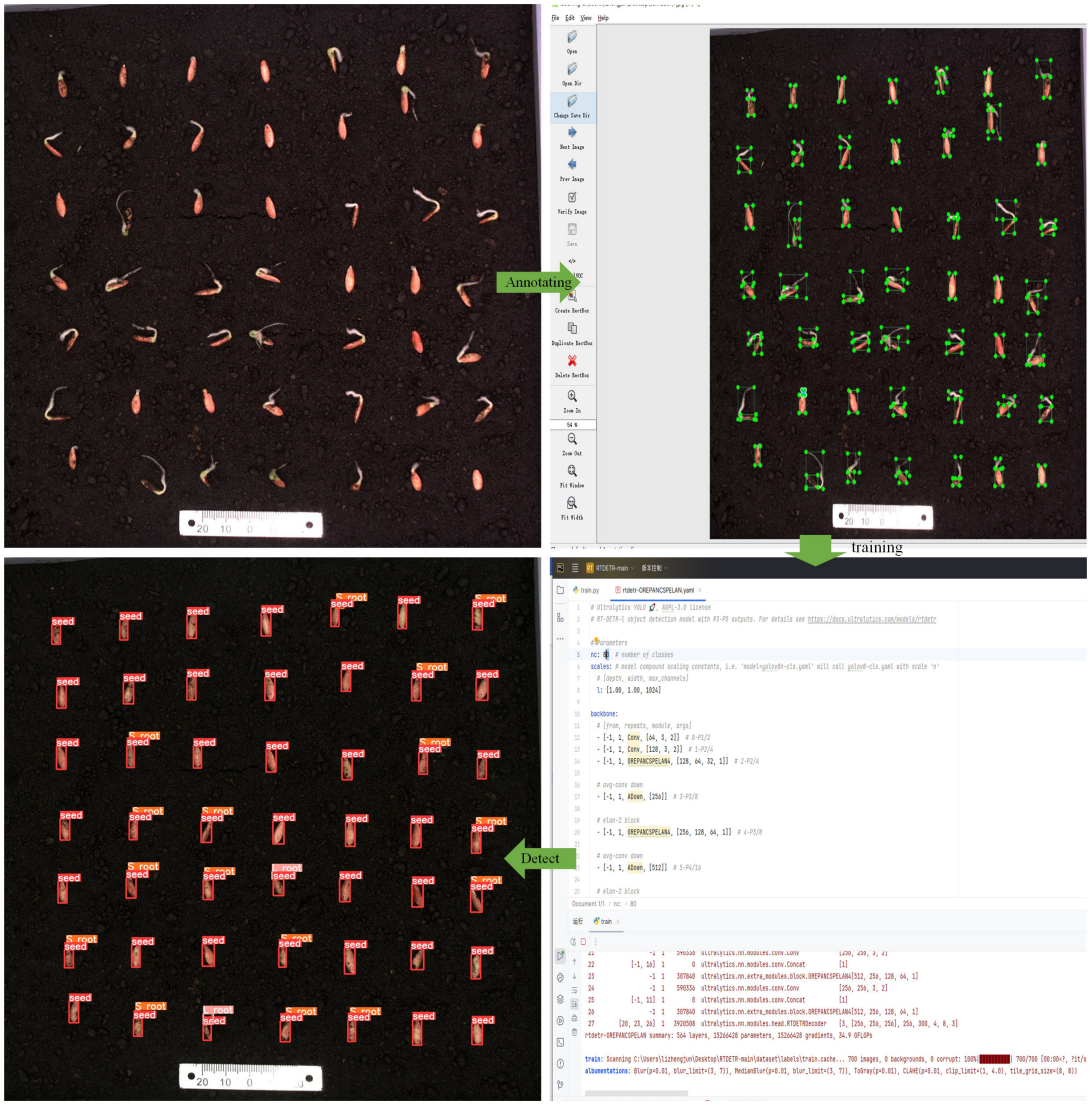
Training process diagram.

The model training environment for this experiment is as follows. Under the Windows 10 operating system, the processor is Intel(R) Core(TM) i5-9300H @ 2.4GHz, and the graphics card is NVIDIA GeForce GTX1650 with 16 GB of RAM. The model runs on Python 3.8 and PyTorch 2.2.1, utilizing GPU acceleration for training with CUDA version 11.8. To avoid the common bug in the GTX series GPU, the cache is set to false. All other parameters are kept at their default values. The training set is randomly divided into training, testing, and validation sets at a ratio of 7:2:1. To ensure a true and effective comparison of performance between different models in all experiments, all models are set to the parameters shown in **Table 1**, and pre-trained weights are uniformly not used to improve accuracy. The image size is uniformly cropped to 640 × 640 pixels, and the number of images per training batch is 4. The training process diagram is shown in **Figure 9**.

**Table 1:** Model hyperparameter settings.

Parameters	Setup
Epoch	100
Batch size	4
GIoU	0.5
Image Size	640 × 640
Cache	False
Workers	4

### 3.2 Ablation experiments

**Figure 10:**
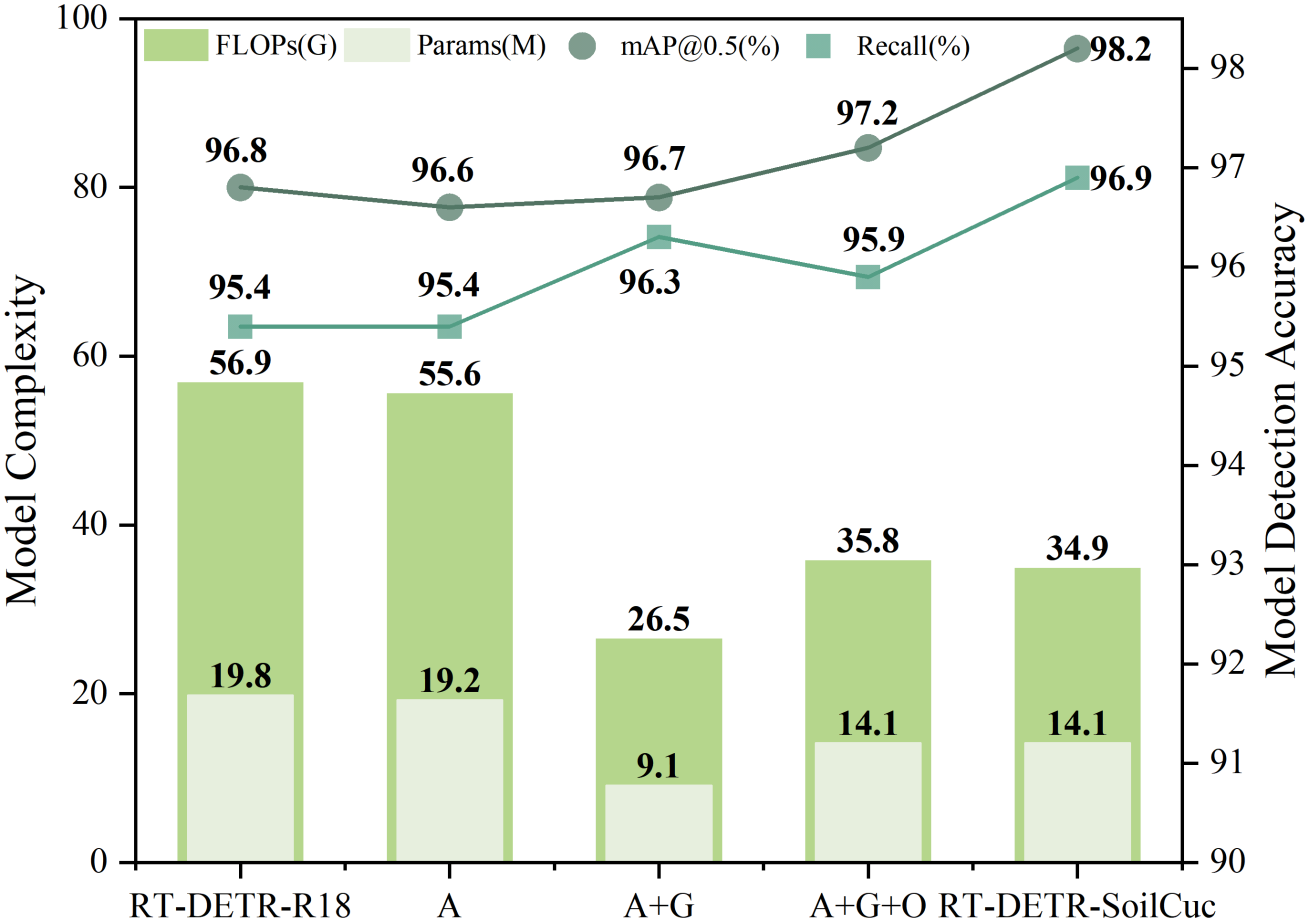
Partial comparison of RT-DETR-SoilCuc ablation test performance indicators. In the figure, A represents ADOWN, G represents GELAN, and O represents OREPA.

Using a randomly selected annotated dataset, ablation experiments were conducted to evaluate the lightweight outcomes, as shown in **Table 2**, and the optimized accuracy results, as presented in **Table 3**. Initially, the most lightweight model in RT-DETR, RT-DETR-R18, was used as the baseline model for enhancement. After introducing the Adown downsampling operator into RT-DETR-R18, the Params, FLOP, and weight size decreased by 3%, 2%, and 3.3%, respectively. However, the three average pooling operations performed by Adown in the backbone blurred the feature information in training images, impacting the model’s ability to locate and recognize embryo root targets, leading to a 0.2% decrease in mAP@0.5, which was undesirable. Therefore, the backbone part of the baseline model was replaced with the GELAN module to enhance the model’s ability to extract and recombine target features. This led to a 0.1% increase in mAP@0.5 accuracy while significantly reducing Params, FLOP, and weight size by 52.6%, 52.3%, and 52.2%, respectively, thus significantly improving the model’s lightweighting level. However, the accuracy was still not ideal. Further, OREPA mechanism was added to the model backbone, where its unique linear scaling layer and module compression could suppress feature interference from the soil-cultivation environment. This resulted in an increase in mAP@0.5 and mAP@0.5-0.95 to 97.2% and 72.3%, respectively. Compared to the previous experiment, Params, FLOP, and weight size increased by 54.9%, 25.9%, and 67.4%, respectively, but the model still maintained a lightweight advantage of around 30% compared to the baseline model. Finally, the NWD loss function was introduced, which, benefiting from NWD’s ability in pixel-level recognition of small targets, improved the model’s ability to distinguish small embryo root targets. This resulted in an increase in mAP@0.5, mAP@0.5-0.95, precision, and recall by 1%, 2.1%, 0.9%, and 1%, respectively, without increasing the model’s Params, FLOP, and weight size. All of the improvements have been completed, and the final RT-DETR-SoilCuc model demonstrates comprehensive advantages over the baseline model under the experimental conditions of this paper. Compared to RT-DETR-R18, its Params, FLOP, and weight size have decreased by 28.8%, 38.7%, and 23.1%, respectively, whereas mAP@0.5, mAP@0.5-0.95, precision, and recall have increased by 1.4%, 3.5%, 1%, and 1.5%, respectively. The intuitive comparison between various parameters in the ablation experiments is shown in **Figure 10**. The fps has reached 24.1, indicating that RT-DETR-SoilCuc can make approximately 24.1 inferences per second, which represents the number of predictions that the model can make in 1 s. Because seed germination is a time-consuming process, the real-time detection capability of RT-DETR-SoilCuc at 24.1 inferences per second meets the demand for detecting cucumber seed germination while outperforming the baseline model in terms of accuracy and lightweight design. This demonstrates the potential of the proposed approach for cucumber seed germination detection in soil-based environments.

**Table 2:** Results of ablation experiments in terms of model lightweighting.

Model	mAP@0.5 (%)	Params (M)	FLOPs (G)	Weight size (MB)	FPS
RT-DETR-R18	96.8	19.8	56.9	38.6	22.2
Adown	96.6	19.2	55.6	37.3	23
Adown + GELAN	96.7	**9.1**	**26.5**	**17.8**	**28**
Adown + GELAN + OREPA	97.2	14.1	35.8	29.8	24.5
RT-DETR-SoilCuc	**98.2**	14.1	34.9	29.7	24.1

Bold indicates the best experimental results.

**Table 3:** Results of ablation experiments in terms of model detection accuracy.

Model	mAP@0.5 (%)	mAP@0.5-0.95 (%)	Precision	Recall
RT-DETR-R18	96.8	70.9	96.4	95.4
Adown	96.6	71.2	95.7	95.4
Adown + GELAN	96.7	71.4	96	96.3
Adown + GELAN + OREPA	97.2	72.3	96.5	95.9
RT-DETR-SoilCuc	**98.2**	**74.4**	**97.4**	**96.9**

Bold indicates the best experimental results.

### 3.3 Comparison experiments

**Figure 11:**
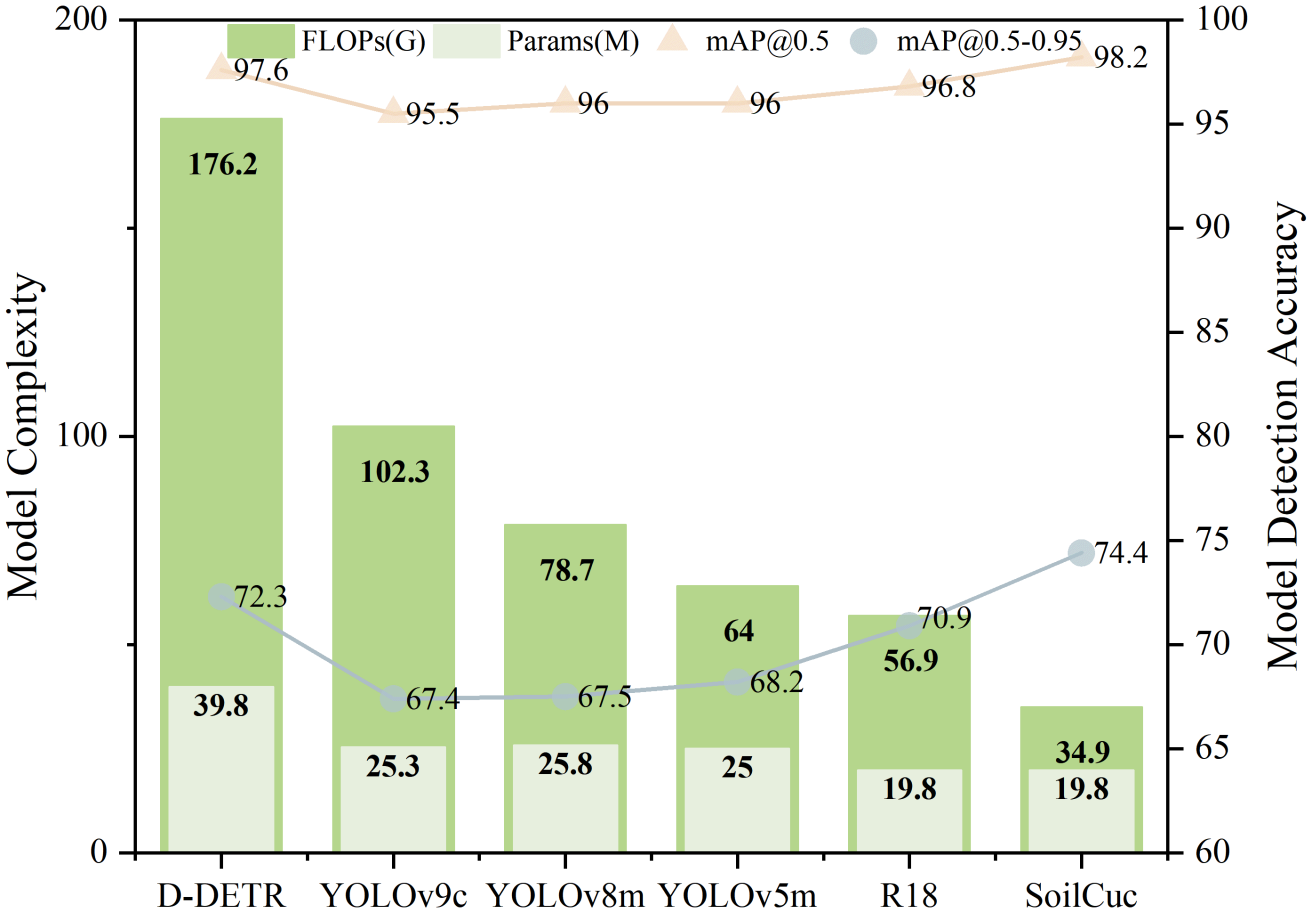
Partial comparison of different models’ performance indicators.

As shown in **Table 4**, RT-DETR-SoilCuc is compared with YOLOv5m, YOLOv8m, YOLOv9c, RT-DETR-R18, and the classic Deformable-DETR model in terms of accuracy and lightweighting metrics (Zhu et al., 2021). The parameters of each model, as shown in **Table 4**, mainly compare mAP@0.5, mAP@0.5-0.95, Params, FLOP, and weight size. The intuitive comparison between various parameters in the comparison experiments is shown in **Figure 11**. It is evident that, without any enhancements, RT-DETR exhibits an advantage in recognition accuracy over YOLO. This is one of the reasons why RT-DETR was selected as the baseline model instead of YOLO. In terms of model lightweighting, RT-DETR-SoilCuc has a significant advantage in lightweighting compared to models of similar size, with reductions of 28.8%, 38.7%, and 23.1% in Params, FLOP, and weight size, respectively, compared to the baseline model. Using model accuracy as the benchmark, RT-DETR-SoilCuc still maintains a significant accuracy advantage, indicating that the model has achieved a relatively optimal level in terms of deployability and recognition accuracy.

**Table 4:** Results of each indicator for different models.

Model	mAP@0.5 (%)	mAP@0.5-0.95 (%)	Params (M)	FLOPs (G)	Weight size (MB)
Deformable-DETR	97.6	97.9	39.8	176.2	456.7
YOLOv9c	95.5	86.0	25.3	102.3	49.2
YOLOv8m	96	74.4	25.8	78.7	49.6
YOLOv5m	96	96.6	25	64	48.2
RT-DETR-R18	96.8	97.5	19.8	56.9	38.6
RT-DETR-SoilCuc	**98.2**	**98.8**	**14.1**	**34.9**	**29.7**

Bold indicates the best experimental results.

**Figure 12:**
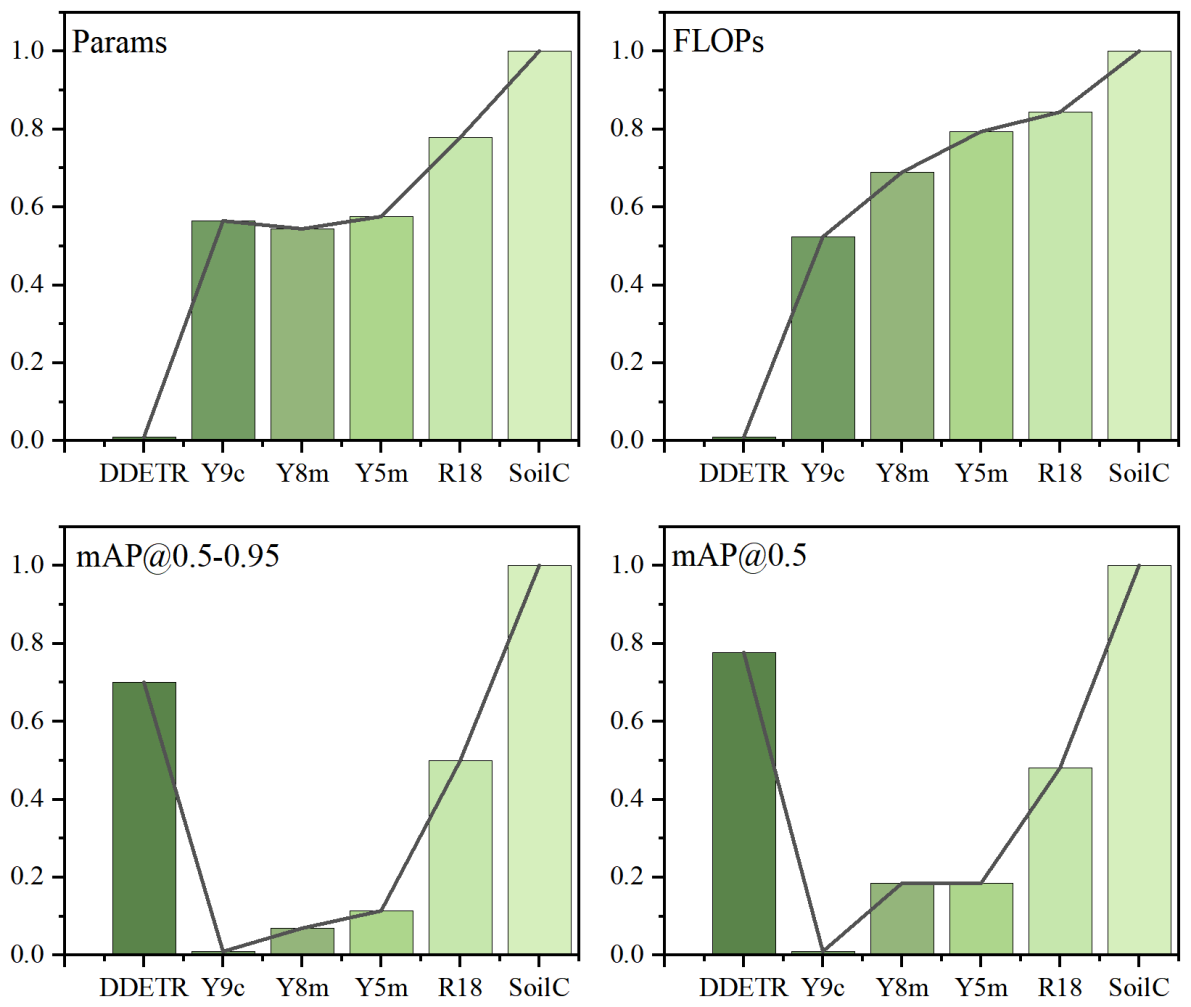
Multi-indicator normalized analysis.

To facilitate a more intuitive comparison of the performance of each model, the parameters of each model were normalized. This involved mapping the accuracy metrics, mAP@0.5 and mAP@0.5-0.95, to a range between 0 and 1. Additionally, the Params, FLOP, and weight size were reverse-mapped, such that lower values corresponded to higher scores. **Figure 12** depicts the normalized histograms of the parameters for each model.

### 3.4 Detection of salt-alkali tolerance in cucumber seeds grown in soil

During seed germination, high-salt environments can disrupt the osmotic balance within the seeds, leading to ion toxicity and potentially resulting in seed death (Zhou et al., 2021). In response to this breeding issue and to validate the performance of RT-DETR-SoilCuc in practical experiments, a salt tolerance experiment was conducted on cucumber seeds in soil using the Zhi Lv 0116 variety as an example.

**Figure 13:**
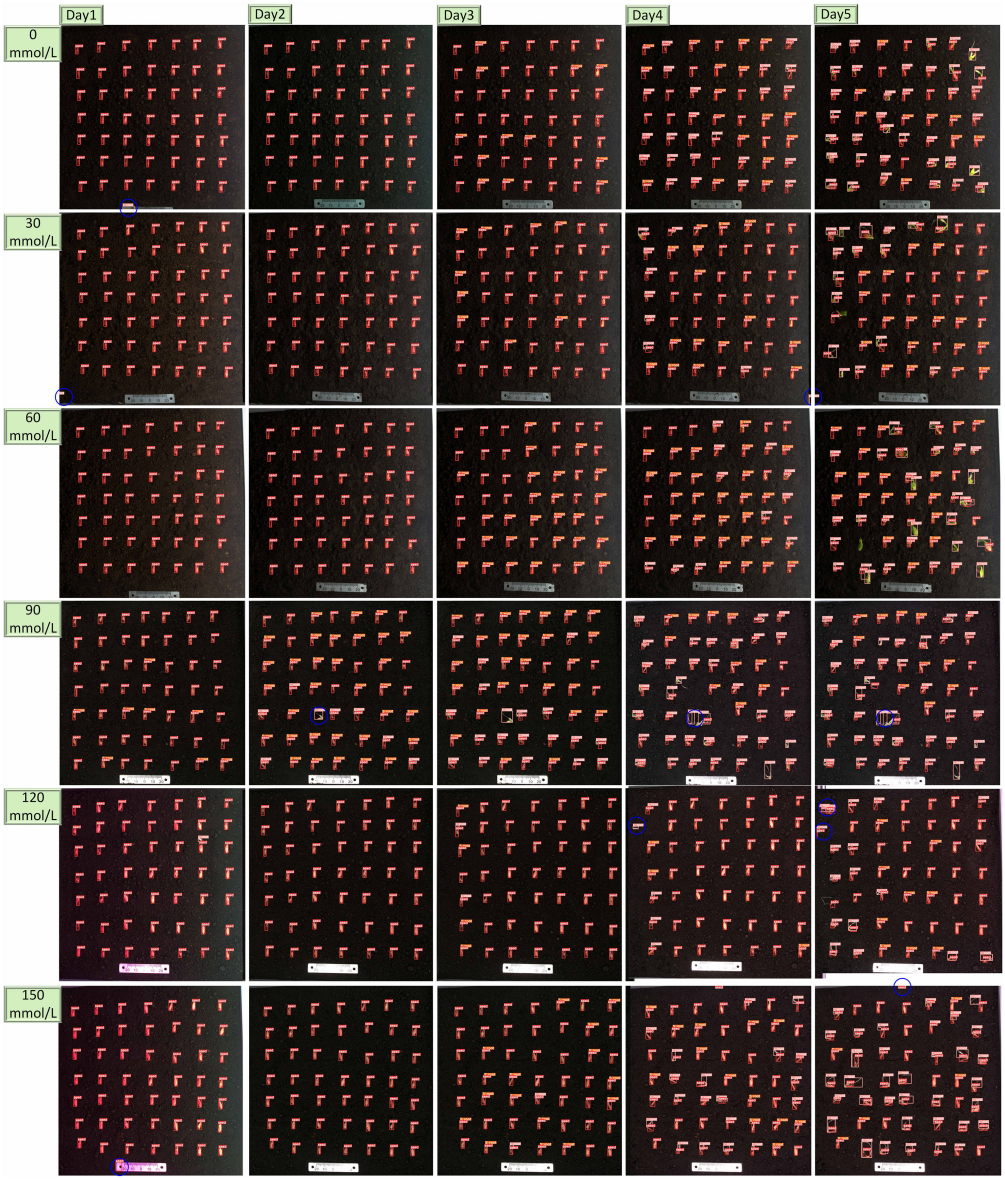
Detection results of RT-DETR-SoilCuc for different growth conditions of cucumber.

Over a period of 5 days, the germination rate and growth vitality of cucumber seeds under salt stress were monitored in real-time using RT-DETR-SoilCuc. Salt stress was simulated with sodium chloride solutions of 0 mmol/L, 30 mmol/L, 60 mmol/L, 90 mmol/L, 120 mmol/L, and 150 mmol/L, with sterile horticultural soil used as the growth substrate. The recognition results of RT-DETR-SoilCuc are shown in **Figure 13**.

**Figure 14:**
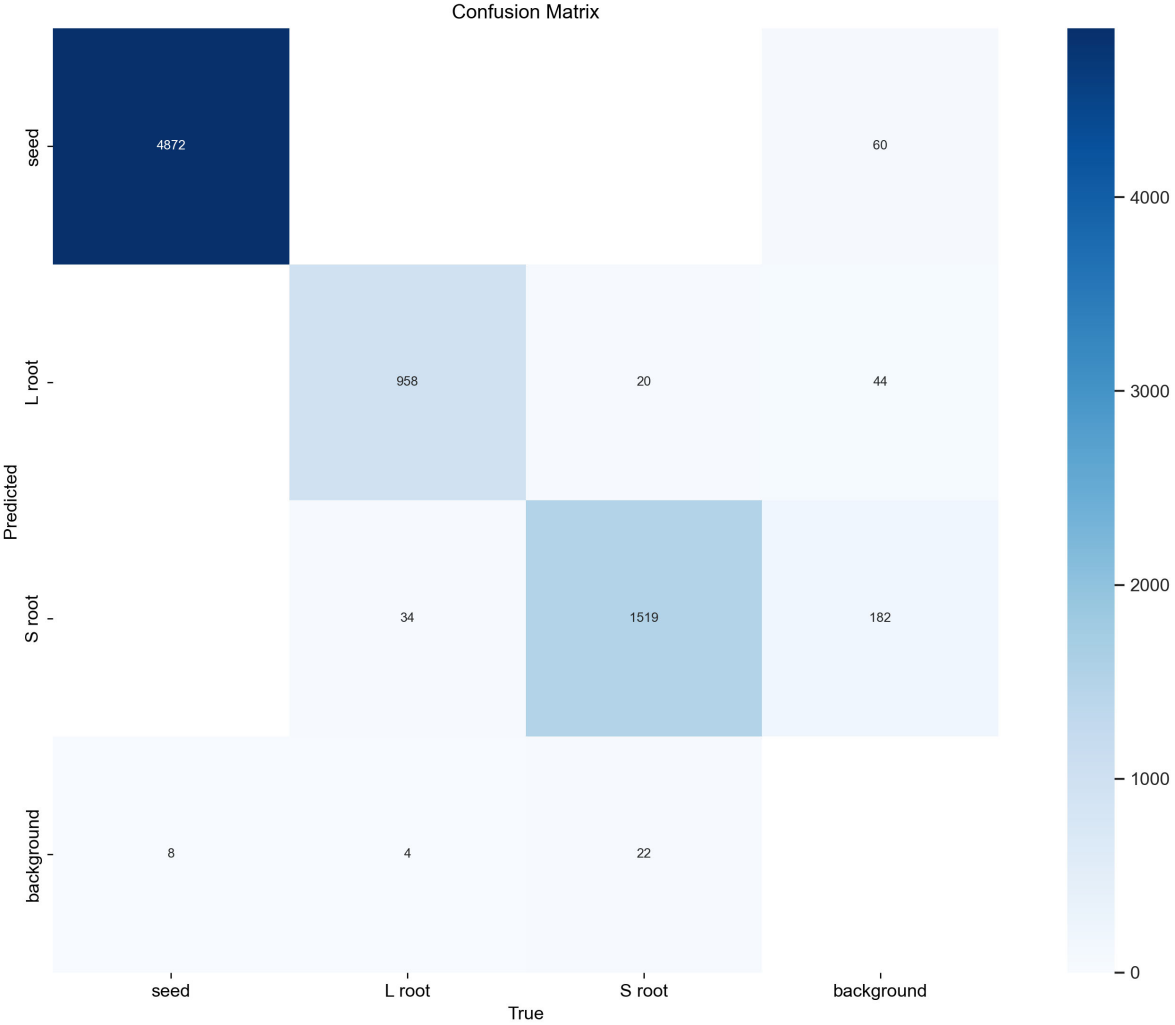
Confusion matrix.

The detection errors, including missed detections, false alarms, and repeated detections, are highlighted with blue circles in **Figure 13**. Among the 30 sample images, a total of 11 detection errors were observed, comprising one missed detection, seven false alarms, and three repeated detections. Considering that each image contains 49 seed samples, the overall detection error rate is approximately 0.78%. **Figure 14** shows the confusion matrix generated after batch processing 100 images for recognition. As depicted in **Figure 14**, background interference is the most significant factor affecting recognition, with the primary recognition error being false alarms. It is anticipated that, by retaining only the soil environment during image cropping, the probability of false alarms can be significantly reduced.

**Figure 15:**
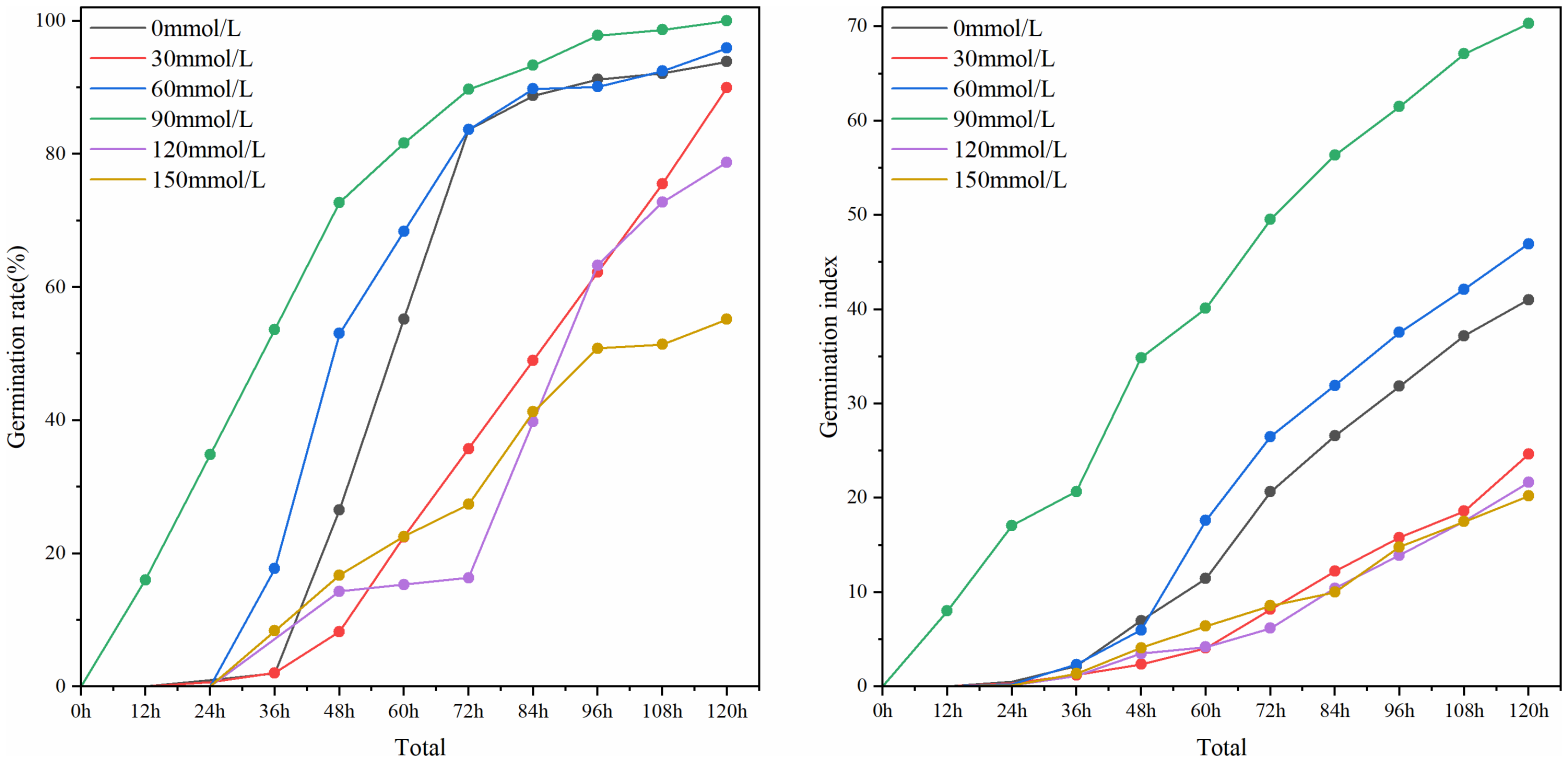
The germination rate and germination index of cucumber seeds under different salt stress conditions.

Based on the detection results of RT-DETR-SoilCuc, plot line graphs in **Figure 15** depict the evaluation of seed germination rate and germination index, respectively.

From the figure, it can be observed that the Zhi Lv 0116 variety exhibits good resistance to low-concentration salinity and alkalinity. Under sodium chloride concentrations ranging from 0 mmol/L to 90 mmol/L, both the germination rate and germination index of this variety increase as the salt concentration rises, indicating a positive effect of low salt concentration on the germination vigor of this cucumber variety. The culture dish with a concentration of 30 mmol/L is positioned near the hot air outlet (as shown in **Figure 1**), which may lead to excessive drying of the moisture, thereby negatively impacting the germination rate and seed vitality. The germination vigor of this variety peaks at a sodium chloride concentration of 90 mmol/L. However, at salt concentrations of 120 mmol/L and above, the germination rate and germination index of Zhi Lv 0116 rapidly decline, suggesting its poor tolerance to higher concentrations of saline-alkali environments. In summary, Zhi Lv 0116 is suitable for cultivation in soil environments with no or low salinization. Its seeds would lose most of their vigor under high salt stress, making it unsuitable for cultivation in highly saline-alkali soil environments.

This experiment verifies the effectiveness and accuracy of RT-DETR-SoilCuc in detecting cucumber germination rates in soil-based work, demonstrating the potential for deployment and use of this model in experimental environments.

## 4 Summary, limitations, and future work

In order to evaluate the germination rate of cucumbers in a soil-based environment that is closer to the actual growth conditions of the seeds and to assist breeders in selecting cucumber varieties with stronger stress resistance, this study addresses the limitations of traditional manual germination detection methods, which are costly, time-consuming, and prone to errors. Furthermore, deep learning–based germination detection methods optimized for hydroponic environments may face challenges in accurately identifying germination in soil environments. To overcome these challenges, we constructed a cucumber germination dataset in soil-based environments using the Seed Germination Phenotyping System. Considering the deployment requirements in low-computing environments, we proposed a real-time cucumber germination detection model, RT-DETR-SoilCuc, based on the soil-cultivated cucumber germination dataset. Compared to other models of similar size, this model is characterized by its lightweight nature and high accuracy. Specifically, we introduced online image augmentation techniques based on the Albumentations library into the baseline model RT-DETR. Subsequently, we replaced different convolutional layers in the backbone of the model with the ADown downsampling operator from the latest YOLOv9 and the GELAN module. We incorporated the OREPA mechanism in GELAN and added the NWD loss function at the end of the model. As a result, RT-DETR-SoilCuc achieved mAP@0.5, mAP@0.5-0.95, precision, and recall rates of 98.2%, 74.4%, 97.4%, and 96.9%, respectively. Additionally, the Params, FLOP, and weight size decreased by 28.8%, 38.7%, and 23.1% compared to the baseline model.

The performance of the RT-DETR-SoilCuc model was compared with the classic Deformable-DETR model and other YOLO series models in terms of lightweight design and deployment. The RT-DETR-SoilCuc model demonstrated higher efficiency with an FPS of 24.1, making it suitable for continuous detection in laboratory settings.

The model’s effectiveness was validated by conducting a cucumber seed germination experiment under salt stress conditions. Results showed that the Zhi Lv 0116 cucumber variety exhibited excellent adaptability to low salt concentrations, with appropriate levels promoting germination while high salt concentrations led to reduced vigor. This experiment verifies the ability of RT-DETR-SoilCuc to detect cucumber seed germination in soil-based environments.

Although the RT-DETR-SoilCuc model achieved its design goals, it has limitations in detecting complex embryonic root targets during later stages of cucumber seed germination. To address this, we plan to optimize the model by establishing datasets using semantic segmentation for deeper analysis of cucumber growth processes.

Our future work aims to enhance the model’s capabilities for analyzing cucumber growth processes and provide valuable insights for soil-based breeding efforts. We hope that the RT-DETR-SoilCuc model can provide convenience for other scholars’ cucumber soil-based breeding work.

## Data Availability

The raw data supporting the conclusions of this article will be made available by the authors, without undue reservation.
